# Integrated Simulation and Calibration Framework for Heating System Optimization

**DOI:** 10.3390/s24030886

**Published:** 2024-01-29

**Authors:** Kirill Djebko, Daniel Weidner, Marcel Waleska, Timo Krey, Sven Rausch, Dietmar Seipel, Frank Puppe

**Affiliations:** 1 Chair of Computer Science VI: Artificial Intelligence and Knowledge Systems, Julius-Maximilians-Universität Würzburg, Am Hubland, 97074 Würzburg, Germany; daniel.weidner@uni-wuerzburg.de (D.W.); marcel.waleska@uni-wuerzburg.de (M.W.); dietmar.seipel@uni-wuerzburg.de (D.S.); frank.puppe@uni-wuerzburg.de (F.P.); 2 ENER-IQ GmbH, Leightonstraße 3, 97074 Würzburg, Germany; timo.krey@eneriq.com (T.K.); sven.rausch@eneriq.com (S.R.)

**Keywords:** automatic calibration, simulation, heating system optimization, genetic algorithm, microservices

## Abstract

In a time where sustainability and CO2 efficiency are of ever-increasing importance, heating systems deserve special considerations. Despite well-functioning hardware, inefficiencies may arise when controller parameters are not well chosen. While monitoring systems could help to identify such issues, they lack improvement suggestions. One possible solution would be the use of digital twins; however, critical values such as the water consumption of the residents can often not be acquired for accurate models. To address this issue, coarse models can be employed to generate quantitative predictions, which can then be interpreted qualitatively to assess “better or worse” system behavior. In this paper, we present a simulation and calibration framework as well as a preprocessing module. These components can be run locally or deployed as containerized microservices and are easy to interface with existing data acquisition infrastructure. We evaluate the two main operating modes, namely automatic model calibration, using measured data, and the optimization of controller parameters. Our results show that using a coarse model of a real heating system and data augmentation through preprocessing, it is possible to achieve an acceptable fit of partially incomplete measured data, and that the calibrated model can subsequently be used to perform an optimization of the controller parameters in regard to the simulated boiler gas consumption.

## 1. Introduction

The operation of heating systems consumes high amounts of energy and therefore implies non-negligible costs. Furthermore, there is no guarantee that a functioning heating system operates optimally. The efficiency of a heating system depends significantly on the configuration of the controller parameters, which is usually performed during initial installation or during maintenance. The quality of these configurations may vary as a common quality metric is simply ensuring that the heating system suffices the minimum quality standards of operation, which is to say, reaching the desired heating temperature and the required hot water temperature to prevent legionella. Beyond this point, optimization is based on the knowledge and expertise of the technician performing the installation or maintenance and is often performed in a heuristic rule-based manner. The input for these rules mostly consists of information that is coarse and qualitative in nature. The optimizations are generally limited by the sensory information available to the technician. In the common case, this information stems from the limited number of sensors of the controllers of the heating system, thus requiring a rather primitive trial-and-error process. This approach to optimization could be improved by using a digital twin, a simulation model, to predict the effects of the modified controller parameters, thus leveraging the engineer’s knowledge more efficiently. This becomes especially useful when parameter changes may significantly affect the operation of a heating system, are not instantaneous, or may disturb residents. The prerequisite for this, however, is a simulation and calibration system. The goal of calibration is to find a set of parameter values, so that the model’s behavior reproduces the real systems behavior to a satisfying degree. Calibration is usually performed manually in a trial-and-error fashion by the systems engineer. Initially, the engineer picks a set of parameter values considered plausible, based on their expertise and knowledge of the system. These parameters are then refined iteratively until a result is obtained that is deemed sufficiently good, with respect to a quality metric such as the Root Mean Squared Error (RMSE) between the measured and simulated data. The quality of the calibration heavily depends on the engineer’s abilities, expertise, system knowledge, and expended time [1,2]. Due to the time consuming nature of selection and manual refinement of the parameter values, the total number of values that can be explored is limited. Additional factors, like non-linear interdependence of multiple parameters, hinder the calibration process further, as multiple parameters may have to be adjusted simultaneously to achieve an improvement. For many technical systems, one single calibration over its lifetime is not enough, as physical modifications and component degradation alter its nominal behavior. In order to ensure correct functioning of the system, the model has to be periodically recalibrated. As manual calibration is a highly work intensive and time consuming task, the application of automatic calibration is favorable. Additionally, for the simulation system to be viable, it has to be sufficiently generic, especially in regards to the preprocessing of the measured data, the calibration of the model, and the interfacing with support infrastructure responsible for data acquisition, storage, and processing of the simulated data. Therefore, an additional consideration is enabling the integration into a possible existing monitoring or data acquisition infrastructure and the generalization of the approach to be viable as an extension to real-world monitoring software.

### 1.1. Technical Context

To address these issues, we propose an architecture consisting of a preprocessing module and a module for performing simulation and calibration, both of which have containerized versions which can be deployed as microservices. For the simulation and automatic calibration, a Genetic Algorithm (GA)-based approach was implemented in Java. It uses a GA variant, namely a Cyclic Genetic Algorithm (CGA), which changes its recombination strategy and mutation rate during execution. The preprocessing software was written in Python and enables the augmentation and modification of measured data using predefined configurable preprocessing functions as well as utilizing a Long Short-Term Memory (LSTM) neural network developed using keras/tensorflow with configurable input and output to perform machine learning based preprocessing. The preprocessor as well as the simulator and calibrator are part of the KINERGY-Project (grant number/FKZ: 03EN1011C), which aims to link the preprocessor and simulator and calibrator together with a rule-based Decision Support System (DSS) by a process controller to form a Decision Integration System (DIS). The goal of the project is to automatically acquire measured data of real heating systems, feed this data into a database, and have a process controller periodically command recalibration of the simulation models as well as check the health status of the heating systems based on their data. In case of detected inefficiencies or abnormal behavior, the decision support system is tasked with generating a recommendation in the form of a controller parameter change for improvement, which is forwarded to the simulator to simulate the proposed change. The process controller then evaluates the simulated data before and after the change. If the simulated data, after the change, shows that a possible improvement was found, then the proposed change is accepted and discarded otherwise. The accepted changes are to be checked for plausibility by a human expert in the first step, then applied to the heating system, and finally the resulting change of the physical systems behavior is evaluated. In a later step, when enough confidence in the system is acquired, the human verification step can be skipped. The project aims for the DIS to be able to predict improvements in a qualitative fashion (better/worse characteristics of behavior) using rather coarse and easy to build models. In the context of this paper, “qualitative improvements” refers to quantitative predictions made through simulation, which are interpreted qualitatively to assess “better or worse” system behavior. Thus, comparing the automated approach to the classical one with human interaction, the input to the DIS resembles the input a human expert would receive during optimization while the DIS mimics the human expert.

### 1.2. Background

The optimization of heating systems can be divided into model-free approaches, which commonly employ a form of optimization through fault detection, and model-based approaches utilizing simulation models or digital twins. Recent work for the first branch incorporates the use of neural networks to detect faults of HVAC Systems [3,4]. In contrast, our approach is focused on the latter branch, employing parameter optimization instead, and aims for a general system level optimization utilizing a simulation model, a digital copy of the real heating system. To assess the behavior of a physical heating system using simulation models, both data and a calibration procedure for the model are essential. While the classic approach to calibration involves a labor-intensive trial-and-error procedure, automatic calibration is a technique used across various domains that tries to alleviate the shortcomings of the manual approach. Replacing manual calibration promises a speedup of the calibration procedure and the freeing up of human resources to be utilized elsewhere. Automatic calibration has therefore seen an increase in utilization in various domains. Applications include the automatic calibration of traffic simulations [5,6,7], where parametrized driving behavior is tuned to fit road utilization, the calibration of building energy consumption simulations [8,9], where energy consumption predictions are compared against monthly energy bills, and the calibration of conceptual rainfall-runoff models [10,11,12,13,14,15] where relatively coarse models are used to predict the runoff, given available, often geographically sparse, data of rainfall and potential evapotranspiration. Additional more uncommon applications include the calibration of CMOS device simulations [16] and the calibration of forest growth models [17]. The most popular algorithms in this field are Evolutionary Algorithms like the Genetic Algorithm (GA) [18] and its variants, Shuffled Complex Evolution (SCE-UA) [19], which was specifically developed to calibrate conceptual rainfall-runoff models, Particle Swarm Optimization (PSO) [20], Differential Evolution (DE) [21], and Simulated Annealing (SA) [22], as well as Bayesian Optimization (BO) [23,24]. Comparisons of the algorithms for different simulation models of different computational complexity have shown that no single dominating algorithm exists and the results of the compared algorithms are of similar quality on average. The performance of the respective algorithm is dependent on the model it is used to calibrate [25,26,27,28,29], which is in accordance with the no free lunch theorem [30], stating that for every advantage an optimization algorithm has in one class of problems, it has a disadvantage of equal significance in another class of problems. The comparisons, however, reveal a favorable feature of evolutionary algorithms like the Genetic Algorithm, which is their versatility and scalability in regards to the amount of parameters to calibrate, as well as their stable runtime [31,32,33,34]. The work of [35,36] has shown that neither manual nor automatic calibration is superior and that automatic calibration can produce results of similar quality to the classical manual calibration, thus making automatic calibration a viable approach due to the associated time savings.

### 1.3. Focus and Contribution

This paper focuses on the simulation and calibration aspect. While other optimization methods exist, such as optimization through fault detection, our approach, as mentioned above, focuses on the methodology of parameter adjustments in heating systems with correctly functioning hardware. For evaluation, a coarse model of a real heating system located in a 30-unit multifamily home in northern Germany was built and calibrated. The data used to calibrate the model was taken from sensor measurements from the corresponding real heating system. In the same fashion as the applications cited above, our evaluation incorporates calibration based on measured data, but does not modify the physical system to assess whether changes observed by modifying the model translate into changes of the same magnitude in the physical system. The verification of an accurate translation to a real system is a potentially lengthy and costly process, involving the acquisition of permissions, personnel, and a significant amount of time. Furthermore, such an evaluation would be highly dependent on the utilized model and the available data, while both aspects should be separated from the methodology. As we will outline in this paper, obtaining accurate or even sufficiently complete data is a non-trivial task, especially when older heating systems have to be retrofitted with measurement devices (sensors), as data acquisition is a research topic in itself [37,38,39]. Our main contribution lies in the methodology and the development of a customizable pipeline from measured data to optimized parameters, which is applied to measured data of a real heating system. Furthermore, to the best of our knowledge, calibration-based systems offer little integration into a broader data acquisition framework, that would help to further automate the calibration procedure from initial measurements, over data preprocessing, to the actual calibration. This lack of suitable integration is also addressed by our approach through the employment of an easy-to-deploy micro-service-based architecture.

### 1.4. Structure

In this paper, we first give an overview of the different components of the simulation and calibration system utilized for the automatic calibration of simulation models, as well as the preprocessor, in the system overview section. The materials and methods section covers our acquired data, along with the lessons learned during this process, and gives a description of the model used for our evaluation. In the results section, we give detailed information about the different experiments performed to evaluate our approach, as well as the outcomes. Finally, we discuss our results and give an outlook on the further development of our system into a complete monitoring framework, incorporating a process controller and a decision support system. For a quick overview of this paper, we recommend reading Section 2.3, Section 3.4 and Section 5.

## 2. System Overview

The complete system consists of two independent modules, a preprocessor and a simulator and calibrator module, which can be used locally or deployed as microservices. For the latter case, both applications have containerized versions, which can be deployed, for example, in a cloud environment as independent services.

**Figure 1:**
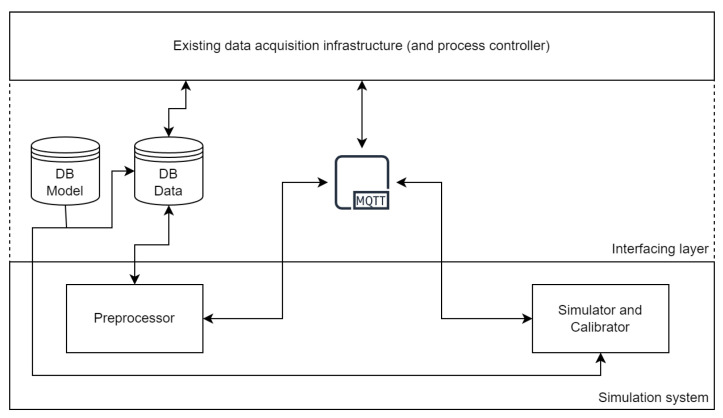
Overview of the microservice system architecture, consisting of an existing data acquisition architecture, an interfacing layer, and the preprocessor, as well as the simulator and calibrator services.

With the increased spread of distributed cloud solutions like Amazon Web Services (AWS) [40] and Microsoft Azure [41], and the associated increasing adoption of containerized microservices in conjunction with container orchestration software like Kubernetes [42], it is natural to enable this type of architecture to a simulation system. Figure 1 shows the architecture of the microservice version. The containerized preprocessor and containerized simulator and calibrator communicate via the Message Queuing Telemetry Transport protocol (MQTT), a lightweight publish-subscribe machine to machine network protocol, and exchange data via a MySQL database, which in turn serves as an interface to existing data acquisition software. The process controller may be included in the existing architecture or as a separate service and is responsible for providing the measured data and sending MQTT commands, as well as for evaluating the output of the simulator and calibrator. This approach enables the automation of the preprocessing-calibration-simulation-evaluation process and would increase the ease of integration into existing microservice-based data acquisition infrastructure, by only deploying a database and a MQTT broker as an interface and an appropriate process controller. For manual use, there is a local version of the preprocessor and the simulator and calibrator. The calibrator features a graphical user interface that can display graphs during calibration. The models and measured data can be supplied in the form of .xlsx- and .csv-files to the local versions instead of a database. In that case, output data is also provided in the form of .csv-files instead of being uploaded to a database.

### 2.1. Preprocessor

When dealing with measured data (in the following referred to as raw data), preprocessing refers to the process of augmenting the data by deriving artificial new features from existing ones and enhancing the quality of the data [43]. This includes the computation of component statuses like the boiler on-off status given its measured gas consumption and the water tapped by the residents given the hot water circuits measured power and volume flows. In general, preprocessing is a time-consuming process commonly delegated as a dedicated task to data scientists. While the initial setup of data preparation most likely will always require manual work, it should be supported by configurable tools. Furthermore, for efficient use within a microservice environment, the adaption of new data should be as seamless as possible, which is to say, the required raw data should be ingested into a storage where it can be processed by the preprocessor and fed back into the same storage, making the result directly accessible for the simulator and calibrator module. To achieve this, a preprocessing application was implemented in Python which can be used as a plain Python application or as a containerized microservice. The preprocessor comes with a set of configurable built-in preprocessing functions, as well as a built-in four-layer keras/tensorflow-based LSTM-network with configurable inputs and outputs. The configuration is obtained via a JSON configuration file, defining inputs and thresholds for the preprocessing functions as well as the inputs and outputs of the LSTM network. The containerized version can be deployed as a microservice and communicates configurations and commands using the MQTT protocol and exchanges data via a MySQL database. The data format is shared by the raw data, the preprocessor input and output, and the simulator input and output. The following operational modes are supported:

- Preprocessing: Raw data → Augmented data
- Reaugmentation: Augmented data → Reaugmented data

The ability to reaugment data allows for preprocessed data variants and fast updates on previously preprocessed data in case only a few features need to be changed. The preprocessor supports the following individually skippable steps:

- Expansion of the timestamp to a (year, month of year, day of month, day of week, hour of day, minute of hour, minutes total) seven-tuple.
- Insertion of missing timestamps and linear interpolation of missing data.
- Extraction of training data using a quality threshold and training of the configurable LSTM network.
- Augmentation of the data with predictions from the LSTM network.
- Augmentation of the data by applying configurable preprocessing functions.

The preprocessor has built-in functionality to extract training data for model calibration from augmented data, by specifying the number of datasets and the number of data points per dataset to be extracted and a quality threshold. Since interpolation of missing data over longer timeframes may have a negative impact on the data quality, this quality threshold is used for training data extraction to ensure that the fraction of interpolated data does not exceed this defined threshold. The preprocessor then tries to extract the specified number of datasets as evenly distributed as possible while satisfying the quality constraint and applies optional configurable (re)augmentation to each training dataset. The resulting augmented data or the (reaugmented) calibrator training datasets are uploaded to the associated database by the microservice version and saved as .csv-files by the local version of the application.

### 2.2. Simulator

The purpose of the simulator is to generate artificial data in order to be able to simulate the effects of changes to controller parameters within the DIS system, and within the context of calibration, to evaluate the fitness function. The simulator and calibrator are written in Java and allow for the definition of component classes with specifiable inputs and outputs. An input can be a data input to either receive external data (measured or augmented data) or simulated data from an output of a different component or a parameter input used to parametrize the model. For each output, it can be defined how the output is calculated as a function of the respective inputs by adding a quantitative description as Java inline-code. This allows for the quantitative descriptions to consist of simple arithmetic calculations or complex structures, using various features of the Java programming language. The component classes can then be instantiated and port-wise interconnected to form the model with the use of a .csv-configuration file. Mappings, given by a .csv-file of sensor names of the measured or augmented data to port names of the model, are used to define which measured or augmented data are to be used. In a similar fashion, value-port mappings to parameter inputs are defined to parameterize the model. To make the model executable, the component classes are compiled, loaded, and instantiated according to the model configuration. The simulation itself is time discrete and conducted on port-level. Iterative, until no output can be simulated anymore, simulatable outputs are determined and their output value is computed by executing their respective quantitative descriptions. These outputs then generate candidates, which are the components connected to them. The outputs of these candidates are considered next for simulation. For the containerized version, the model and configuration data is held in a MySQL database instead of the aforementioned local files.

### 2.3. Calibrator

The calibrator is used to generate values for the model parameters by optimizing the model behavior in regards to a fitness function, which in our case is the RMSE-value between the measured and simulated data, augmented by optional penalty-expressions. The parameters themselves can be divided into “free parameters”, which are calibratable and generally potentially unobservable internal parameters, and controller and settings parameters, which are assigned a fixed value and correspond to controller parameters or features like sensor positions. The automatic calibration itself is performed by a GA variant, namely a Cyclic Genetic Algorithm (CGA) using the jenetics library [44] written in Java. The jenetics library was chosen for its good parallel processing capabilities and modern architecture. The CGA consists of two independently working GAs, the GA Calibrator and the GA Diversifier. Each individual of the population of the GAs is a complete set of parameter values. There is one value for each parameter. The population is iteratively evolved towards solutions, which minimize the RMSE value between measured data and simulated data. The initial population is generated by choosing random values for the parameters, given predefined lower and upper bounds. The population during each iteration is called a generation.

Algorithm 1 shows the pseudo code of the Cyclic Genetic Algorithm. The GA Calibrator receives the initial random population, plus the measured data series of the physical system and the simulation model, whose parameters are to be determined. For each candidate solution in the current population, the fitness function is evaluated. To compute the fitness, a simulation is performed. Each individual of the population is evaluated by using the measured input data, together with the parameter set of the respective individual to generate simulated values and to compute the RMSE fitness value. Since multiple different outputs may be involved in computing the fitness, each output’s influence is equalized. The lower the RMSE value, and therefore the discrepancy between the measured and the simulated data, the higher the fitness of the solution. The computation of the fitness values is parallelized with an adjustable number of parallel threads. Each thread is using a separate instance of the simulator. All simulator instances operate on the shared model and are supplied data in a read-only fashion. The simulator instances are assigned to the different fitness evaluation threads at runtime by a dispatcher. Using the fitness of the current population’s solutions, a new generation of individuals is formed by selection, recombination, and mutation with a low probability. For selection, during each iteration, 40% of the population are picked to be survivors and directly transferred to the population of the next generation. The remaining 60% are recombined and mutated. For mutation, both the GA Calibrator and the GA Diversifier use a gaussian mutator. For recombination, the GA Calibrator uses a variant of mean crossover, where given an individual, the likelihood of selecting a recombination partner *x* is determined by the fitness of *x*. Individuals of similar fitness, which is used here as a proxy for the similarity of parameter values, are more likely to be recombined, hence generating similar offspring which in turn is more likely to be valid. The recombination itself works like simple mean crossover, where random genes (parameter values) are pairwise averaged. The mutation and recombination rates both default to 0.02. Since the changes produced by recombination in combination with a low mutation rate are fine grained and converge over time to what may be a local optimum instead of a global optimum, the GA Diversifier is employed for better exploration of the parameter space. The GA Diversifier uses simple mean crossover as recombination strategy. During execution of the GA Calibrator, when no improvement was made for *i* generations consecutively and therefore the local termination criterion of the GA Calibrator is met, the current population, together with the measured input and output values, is passed to the GA Diversifier for *k* generations, with *i* and *k* being configurable hyperparameters and default to 10 and 15 respectively. The GA Diversifier is using the same population, population size, and fitness function as the GA Calibrator, but has a higher mutation and recombination rate, each having a default value of 0.8. The GA Diversifier is used to help escape local optima, by altering the population stronger than the GA Calibrator. After diversification, when the local termination criterion of the GA Diversifier is met, the current population from the GA Diversifier is passed back to the GA Calibrator. The best individuals, however, are never forgotten due to the selection process, which uses a tournament selector, hybridized with an elite selector, so a diversification step does not decrease the population’s potential fitness. After a diversification, the population is more diverse and the RMSE and parameter values of the individuals are more spread out. If no improvement was made after a diversification phase, the number of iterations the GA Calibrator is allowed to perform before another diversification phase is started, is doubled, causing more fine-grained than coarse-grained changes the further the calibration procedure advances. Whenever an improvement is made, this value is reset to *i* generations. The algorithm terminates when the global termination criterion is met, namely, when a certain number of generations is reached, with the default for the maximum number of generations being 300. Models are calibrated in full with implicit separation of the individual components. During calibration, measured values by default overwrite simulated values, so that models are calibrated purely on component level if all of their input and output data is available, and as component compounds with minimal use of simulated values otherwise. This can be used to reduce the effects from overparameterization, which component-based approaches are prone to, if enough measured data for a sufficient component coverage exists. This default behavior can be modified to allow for the use of simulated instead of measured values, as well as the exclusion of certain values from the fitness calculation via configuration on a per port basis. The first option is useful in cases where, for example, only the temperature of a temperature-flow pair is known and the passing of a measured temperature in conjunction with a simulated volume flow to another component is undesirable as it may distort the resulting computed power. Also, when measured data is rather inaccurate or only sparsely available, it may be beneficial to let the overall model compensate for possible inaccuracies.    
**Algorithm 1:** Cyclic Genetic Algorithm
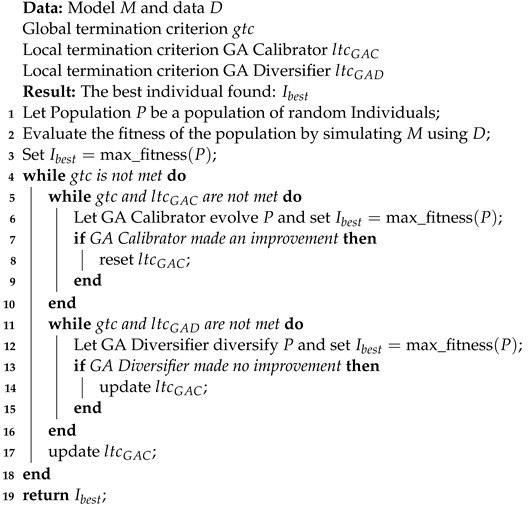


In addition to the use of the calibrator to find parameter values for the free parameters, it can also be used to generate values for the controller parameters. Since this requires the modification of the calibration target, the calibrator allows for the definition of so-called penalty-expressions using a Java-like syntax, which are interpreted by a custom interpreter using the ANTLR parser generator library [45]. It is to be noted that penalty-expressions are not limited to controller parameter calibration but can be used during regular calibration too. In the following, “port mapping” refers to the configuration part that among other settings includes the definition of the sensor-to-port mappings, the value pairs that should be included in the calculation of the RMSE, the measured values which are not allowed to overwrite simulated values, and the penalty-expressions. Furthermore, “parameter mapping” refers to the output of the calibrator, which is a value assignment for each parameter.

The process of generating controller parameter values is described in Algorithm 2.    
**Algorithm 2:** Generate Controller Parameters  **Data**: Model *M* and data *D*  Parameter mapping with fixed controller parameters and calibratable free parameters $c_{param,fpc}$  Port mapping for free parameter calibration $c_{port,fpc}$  Port mapping for controller parameter calibration (with penalty-expressions) $c_{port,cpc}$  **Result**: Set of controller parameter values1Generate parameters $p_{fpc}$ for the free parameters defined in $c_{param,fpc}$ by performing a calibration using *M*, *D*, $c_{param,fpc}$ and $c_{port,fpc}$;2Turn the controller parameters in $c_{param,fpc}$, which are to be calibrated, into free calibratable parameters while keeping the other parameters fixed to the values from $p_{fpc}$ to generate a new parameter mapping $c_{param,cpc}$3Perform a new calibration using *M*, *D*, $c_{param,cpc}$ and $c_{port,cpc}$ to generate parameters $p_{cpc}$ for the selected controller parameters4**return** $p_{cpc}$

The fitness metric for the calibration using penalty-expressions is the sum of the RMSE of the specified output ports in the respective port mapping and the evaluated penalty-expressions, e.g., Penalty-Expression 1 gives a high penalty when, given the current parameters, the simulated hot water temperature falls below 45 °C or exceeds 75 °C.


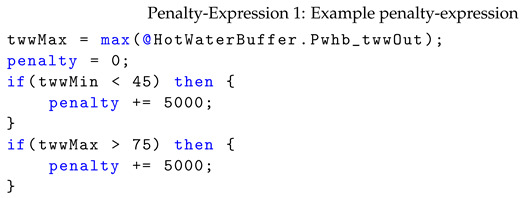


References to all values for a given port for a single dataset are provided using “@” for simulated values and using “$” for measured values. Additionally, using “&”, it is possible to reference parameter values. Each penalty-expression has to assign a value to the variable “penalty” and is evaluated independently for each training dataset during calibration. In case of the example from Penalty-Expression 1, for each dataset independently, the expression is evaluated on all simulated values of port Pwhb_twwOut of component HotWaterBuffer.

The simulator and calibrator can be run as a plain Java application or as a containerized microservice. The containerized version, just like the preprocessor, communicates configurations and commands via MQTT and is supplied with models and measured data via a MySQL database. The resulting calibrated parameter sets and simulated data is uploaded to the same database or saved as a .csv-file for the local version.

## 3. Materials and Methods

In order to perform the calibration of a simulation model, a sufficient amount of high quality data is required. The quality of the data generally correlates with the quality of the predictions derived from the model. Noise in the data may lead to over fitting while missing data may introduce skews into the model’s behavior, e.g., when data for an operational mode is missing entirely, the model’s behavior may show arbitrary discrepancies for this mode, depending on the technique used to derive the model behavior. In practice, the availability of data is a major hurdle when applying data driven approaches to real world scenarios. Noise and temporary outages of the data acquisition systems (IoT sensor connection, power supply, maintenance, etc.) are to be expected. Additionally, some types of data, like resident behavior, is often difficult to obtain at all due to privacy concerns and/or general refusal of the residents to have sensors installed in their apartments. A central aspect of the work presented here is to evaluate the feasibility of calibrating and optimizing a system on a qualitative level, in the absence of direct resident data based on qualitative indicators of better/worse system behavior, given quantitative output of the simulation model. In the following section, first the available data is discussed and then the major hurdles we faced regarding data acquisition are described.

### 3.1. Data Acquisition

Measured data was collected within the test property, a 30-unit multifamily apartment building, built in 1985 and modernized in 2017, with a heated area of 1.816 m^2^, in northern Germany, using various technologies. The recording of temperature data was made possible by retrofitting the heating system with temperature sensors (PT1000 class B, approx. ±(0.30 °C + 0.005|t|) error). These were installed either as contact temperature sensors or, if available, in an immersion sleeve. Heat quantities, power outputs, volume flows, and also temperatures were recorded by Diehl Metering Sharky 775 heat meters (approx. ±1% to ±1.8% heat quantity error, depending on the flow rate). The measured data was recorded by a permanently installed data logger (Lertes; type RmCU) with analog input module (MBS, AI-24) with a time resolution of one minute. Heat meters were read out via the meter bus (M-Bus) and the gas meter pulses were recorded by an IN-Z62 (Elster). The required counter was located in the data logger. The outside temperature was supplied in a 20 min interval, as opposed to the one minute interval for the other data, by a nearby weather station. The recorded data was encrypted using SMIME and sent via email to the ENER-IQ GmbH data ingestion infrastructure. As there was no landline internet access on site, data was transferred via a mobile communication network.

The received emails were decrypted within the ENER-IQ data ingestion infrastructure and processed for storage in the internal time series database. The system component configurations (e.g., boiler, heating circuit, etc.) were set via a user interface. These configurations included controller parameters and characteristic curves as well as technical features. Furthermore, the semantic assignment of the transmitted data points to the system components (sensor assignment) was defined. Finally, the time series were processed and aggregated in order to better visualize larger time periods via the user interface.

### 3.2. Data

Effective system design work started in February 2020 and was therefore set as the date of reference. Available data was retrieved for one year periods with new data being acquired periodically during the course of the project. A complete set of controller parameters, however, could only be acquired for the February 2019–February 2020 period, as the web-interface to read critical parameters became unreachable some time after February 2020 and no direct access to the physical boiler could be granted. The available data and the availability of the controller parameters is listed in Table 1.

The datasets featured varying amounts of missing data, stemming in part from outages of the mobile network connection used to transmit the data as well as downtime due to regular and unscheduled maintenance of physical infrastructure and updates to the data acquisition backend. Since the contact temperature sensors were attached externally to pipes and therefore affected by the sensor position as well as the material and insulation of the respective pipes, we did not perform a further uncertainty analysis of the measured data. However, we conducted quality checks through manual inspection of the acquired data. An issue encountered during data acquisition was the media break when accessing the controller parameters of the physical components of the heating system. Older heating systems tend to be analog focused with often little options to remotely extract the controller parameters. Configuration is usually performed via a terminal with a display and a set of buttons on site. The retrofitting of these systems is therefore bottlenecked by these analog controllers. Acquisition of the controller parameters further suffers an incalculable time delay as access to the physical heating system is dependent on the availability of the respective owner, janitor, or technician responsible for maintenance. Further, there is always the risk of the acquired parameters moving out of sync with the actual ones due to undocumented changes performed as a result of maintenance or repair work. For the datasets except the 2019–2020 timeframe, the controller parameters for the boiler were unavailable, unfortunately, since the web-interface to read those parameters became unreachable some time after February 2020 and no direct access to the physical boiler could be granted. The remaining parameters were collected by photographing the controller displays. The 2020–2021 timeframe was dropped from the evaluation, since it neither featured recent boiler controller parameters nor have there been parameters of the other controllers available for that time period. For the 2021–2022 dataset, initially the missing on-off threshold boiler parameters were estimated with the help of a domain expert and made calibratable parameters within a tight range of the expert estimates, however, the high amount of missing data as well as an unusual parameter modification (doubling of the boiler pump volume flow and increase in the heating circuit flow temperature to 70 °C) up to 12 days into the measurement period, likely stemming from an undocumented modification, proved to be a hindrance during the extraction of suitable training data for the whole timeframe, as each training dataset would have needed to contain up to 20% interpolated data. The 2019–2020 dataset, on the other hand, featured the best quality overall as the most controller parameter information was available and the amount of missing data was the lowest. Since additionally no reliable controller parameters could be obtained after February 2020, the 2019–2020 dataset was chosen for all subsequent evaluations. The dataset was preprocessed by the preprocessor pipeline as laid out in the preprocessor section. First by interpolating missing data, then training an LSTM network to predict the heating circuits power and the volume flow of the heating circuits pump, given the features month of year, weekday, hour of day, minute of hour, and outside temperature. While the heating circuit power and volume flow was available as measured data, the LSTM predictions were used instead for the following calibrations and simulations, as the LSTM predictions could be used to fill missing data, which would have otherwise needed to be interpolated. Additionally, the LSTM predictions smoothed out occasional spikes. Finally, the datasets were augmented by adding artificial features like the computed amount of water tapped by the residents and the on- and off-state of the boiler. Missing data was linearly interpolated. The resulting preprocessed data was automatically partitioned by the preprocessor into eight training datasets of 5000 consecutive data points, with the constraint that the training datasets had to be picked from the full dataset as equally distributed as possible while the fraction of interpolated data points within each training dataset had to be less than 5%.

### 3.3. Lessons Learned from Data Acquisition

During data acquisition, we encountered systematic challenges, which we think translate to this type of data acquisition in general. In the following, we discuss these challenges and our mitigation strategies.

#### 3.3.1. Retrofitting Heating Systems

Older heating systems especially may have a lengthy history of maintenance procedures, some of which might not have been well-documented, or not documented at all. Generally, the first action to retrofitting the heating system with sensors is therefore an extensive inspection of the physical system and comparison against the hydraulic diagram (if available). From this, an updated hydraulic diagram can be derived which can be annotated with measurements regarding the geometry and available space of the boiler room for the subsequent installation of sensors.

#### 3.3.2. Resident Behavior Data

As the hot water consumption of the residents is a major source of energy consumption, this behavior should be captured whenever possible. Unfortunately, in our case the means to do so were limited, as no data could be obtained from the individual cold water meters and no other suitable sensors could be installed in the residents’ apartments. Consequently, we were unable to capture specific details regarding the residents’ hot water consumption and heating preferences and had to resort to deriving estimates based off the temperatures and volume flows of the heat exchanger of the hot water system. The volume flows on the secondary side of the heat exchanger, however, were also not available initially and had to be obtained on-site with the help of two Taconova TacoSetters [46]. Since the volume flows are also dependent on the potential dynamic charging state of the hot water buffer tank, which in turn is affected by the unknown and estimated hot water consumption of the residents, the missing resident behavior was a major source of inaccuracy. Furthermore, no measurements of the volume flow of the heating buffer tank on the secondary side were available (from/to the storage tank to the hot water system and the heating system). These values, however, were again reliant on the combined behavior of the heating circuit and the hot water circuit, both affected by our estimated resident behavior.

#### 3.3.3. Acquisition of Controller Parameter Data

In order to perform a meaningful calibration and to observe changes to the controller parameters, the initial values for these controller parameters have to be known. For older heating systems, like in our case, this may involve an inspection of the control displays on-site. It is generally advisable not to assume that older heating systems are optimally-maintained. The controller parameter acquisition on-site proved to be unexpectedly challenging as, due to reasons beyond our control, no access to the physical boiler could be granted. The boiler’s controller parameters, however, were in theory accessible via a web-interface, with a highly unreliable connection, which became completely unavailable some time after February 2020.

### 3.4. Model

**Figure 2:**
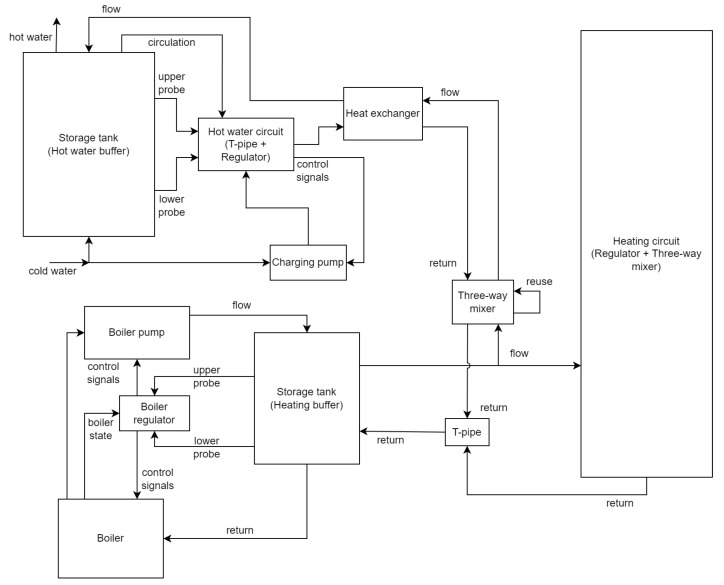
Schematic overview of the heating system model.

After the data acquisition, a coarse simplified model of the corresponding physical heating system was built, consisting of the components “boiler”, “boiler pump”, “boiler controller”, “heating storage tank (heating buffer)”, “t-pipe”, “three-way mixer”, “heating circuit (controller + three-way-mixer)”, “heat exchanger”, “water heating controller (t-pipe + controller)” “hot water storage tank (hot water buffer)”, and “charging pump”. The components are 0D models with the exception of the storage tanks, which are 1D models featuring 20 nodes each, to model the water stratification within the tanks. The choice of 20 nodes was made empirically, considering computational performance factors. The boiler (ELCO Trigon XL 150) has a heat output ranging from approximately 31.4 kW to 142.3 kW, without condensing energy usage, and 35.2 kW to 149.4 kW with maximum condensing energy usage (taken from the product label, photographed during a past inspection, as these values slightly deviate from the current data sheet). The heating buffer (ELCO VISTRON B 400-1) has a volume of 420 l and the hot water buffer (Danfoss ThermoDual S 0300-060 XB37) has a volume of 300 l. The modeling principle behind the storage tanks is comparable to [47], albeit being more heuristic in nature while still featuring simultaneous charging and discharging. The inflow and outflow of components is divided in the primary side, the side towards heat generation, and the secondary side, the side towards heat consumption. Thermal inertia is coarsely accounted for by calibratable rolling means, calibratable cool-down approximations, and custom calibratable processes. These methods are selectively applied to components such as the heating circuit, boiler, t-pipe, and heat exchanger, which use one or more of these methods, depending on the specific characteristics of each component. Additionally, the measured outside temperature is transformed to a damped outside temperature during preprocessing before being supplied to the model. Given that the data has a one-minute resolution, our simulation is also performed with a one-minute time resolution. The model features 128 parameters of which 42 are calibratable. Of the 86 non-calibratable parameters, 33 are for alternative operational modes, for example, parameters for alternative characteristic curves of various components which are not used for the following scenarios and are therefore fixed to an off-state. Another 15 unused fixed value parameters are various scalar and offset parameters, which can be used to adapt the model to systematic input data bias, which were set to 1 and 0, respectively. Generally, it would also be possible to include corrections to bias in the measured data using preprocessing. Additionally, making the aforementioned scalars and offsets calibratable would allow for more dynamic corrections, if needed. The remaining 38 fixed value parameters are controller parameters corresponding to available settings of the physical controllers or parameters to configure component features, like sensor positions. For an overview of the calibrateable parameters, please refer to Appendix A. Figure 2 shows a schematic overview of the model.

## 4. Results

To evaluate the calibrator and simulator, two scenarios were considered, in the following also referred to as “model calibration” and “parameter optimization”. For the first scenario, calibration runs with different configurations were conducted by allowing the calibrator to pick values for the free parameters. The calibrations featured a configuration which let measured values overwrite simulated ones and a configuration that used simulated values exclusively. The resulting calibrated configurations were then compared against the measured data.

In the second scenario, parameter optimization in regards to the heating system’s gas consumption was performed by setting the formerly free parameters to the values obtained from the initial calibration. Additionally, some of the formerly fixed controller parameters were turned into free parameters, and a subsequent calibration of these parameters was carried out. In this scenario, penalty-expressions were used, penalizing a too high volume flow temperature at the heating buffer storage tank secondary side, gas consumption, oscillatory status switches, and too low as well as too high hot water temperatures. All calibrations were performed on the previously extracted eight datasets of 5000 data points each and evaluated on the full 2019–2020 dataset consisting of 525,600 data points.

For a concise overview of the results presented in this chapter, please refer to the summary in Section 5.

### 4.1. Model Calibration

Two types of calibrations were performed. First, a configuration *sim_only_false* was used, which let measured values overwrite simulated ones wherever possible. Subsequently, a configuration *sim_only_true* was employed, which did not allow any measured values to overwrite simulated ones. Using the configuration *sim_only_false*, components whose inputs were exclusively primary measured inputs (e.g., outside temperature) or outputs from other components that were overwritten by measured values were effectively calibrated in isolation. A downside of this configuration is that it prevents the overall model from compensating for inaccuracies of single components. Therefore, when calibrating a model using *sim_only_false* and then using these calibrated parameters for simulation of the *sim_only_true* configuration, the overall performance may be worse compared to a model calibrated using the *sim_only_true* configuration directly. The hyperparameters of the CGA used for the calibration are the default values, as described in Section 2.3. In the following, *sim_only_false* is also referred to as *so_false* and *sim_only_true* as *so_true* for brevity.

### 4.2. Model Calibration Results

In the following, the behavior of the model calibrated using the different configurations is presented graphically, based on snapshots from the full dataset, as well as in terms of the RMSE-values of the key temperatures.

**Figure 3:**
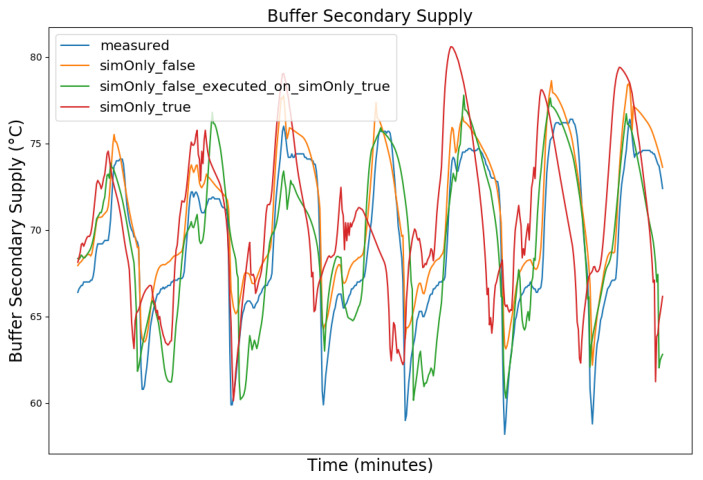
Data snapshot from 27 February 2019 05:00 a.m. to 27 February 2019 01:20 p.m. The graphs are showing the supply temperature of the heating storage buffer tank (secondary side) as a comparison of the measured data (blue), the model calibrated on the configuration *sim_only_false* without simulated values overwriting measured ones (orange), the model calibrated on the configuration *sim_only_false* with simulated values overwriting measured ones (green), and the model calibrated on the configuration *sim_only_true* with simulated values overwriting measured ones (red).

**Figure 4:**
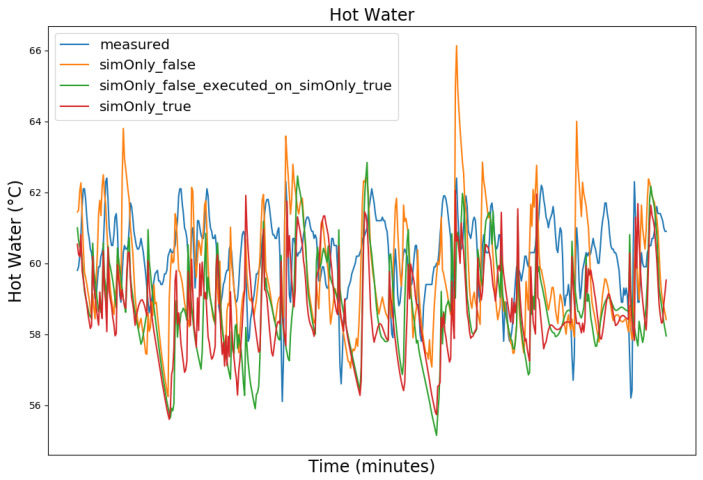
Hot water temperature at the hot water storage tank outlet. For an explanation of the graph legend, please refer to the caption of Figure 3.

**Figure 5:**
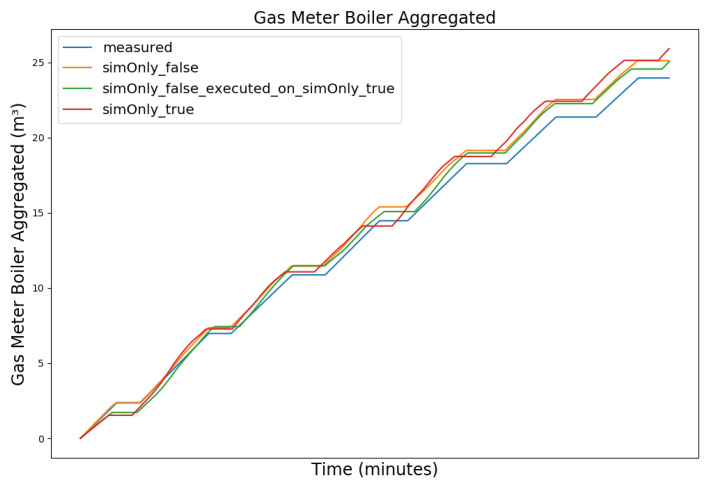
Aggregated boiler gas consumption. For an explanation of the graph legend, please refer to the caption of Figure 3.

**Figure 6:**
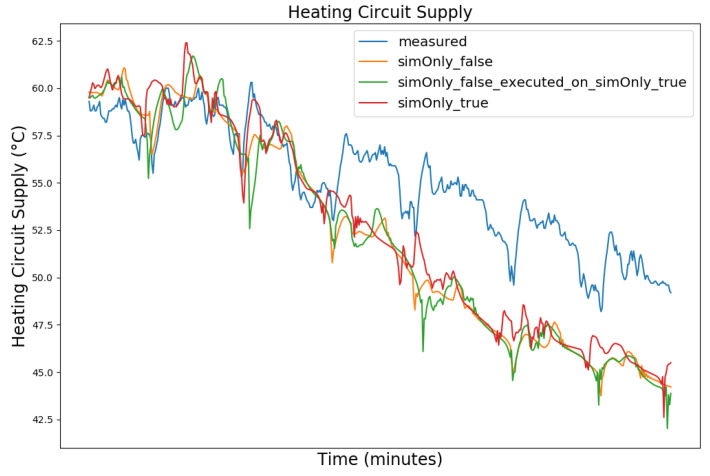
Supply temperature of the heating circuit (primary side). For an explanation of the graph legend, please refer to the caption of Figure 3.

**Figure 7:**
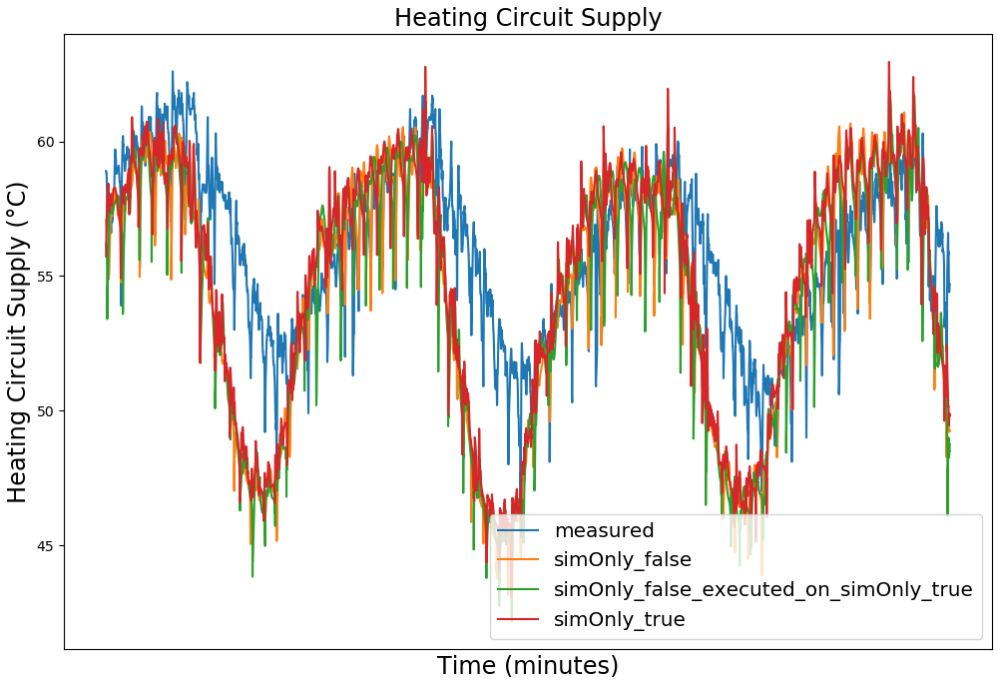
Data snapshot from 23 February 2019 10:40 p.m. to 27 February 2019 10:00 a.m. for the supply temperature of the heating circuit (primary side). For an explanation of the remaining graph legend, please refer to the caption of Figure 3.

Figure 3, Figure 4, Figure 5 and Figure 6 exemplary show the system behavior given by the measured data, as well as the model behavior calibrated on *sim_only_false* and *sim_only_true* for snapshots from the 2019–2020 dataset. For the model calibrated on *sim_only_false*, two runs were performed. The first run (labeled as simOnly_false in the Figure 3, Figure 4, Figure 5 and Figure 6) allowed measured values to overwrite simulated ones, similar to the calibration process, and the second run (labeled as simOnly_false_executed_on_simOnly_true) used simulated values only, mirroring the run for the model calibrated on *sim_only_true*. The model successfully captured the overall behavior qualitatively, but also exhibited deficiencies on a quantitative level, as evident from the graph of the heating circuit supply temperature of Figure 6. Expanding the timeframe, as shown in Figure 7, reveals systematic discrepancies in the simulation that stem from the model itself rather than the chosen parameter values during calibration.

In this particular case, all calibrated models behave similarly, as the heating circuit has few parameters and no intermediate measured values associated with it. However, for our goals, we deem this level of accuracy sufficient, as we are primarily interested in qualitative changes in behavior.

Due to readability reasons, no elaborate graphical overview is presented and instead the focus is set to the key RMSE temperatures, as the RMSE was also the optimization criterion during calibration. The values of the following tables are based on the non-artificial (interpolated) values.

Table 2 shows the comparison of the RMSE values for the key temperatures for the models calibrated using the configuration *sim_only_false* and *sim_only_true*. The variant *so_false* (1) corresponds to the graphs labeled as simOnly_false and the variant *so_false* (2) corresponds to the graphs labeled as simOnly_false_executed_on_simOnly_true. The results indicate overall acceptable errors, especially when considering that temporal shifts are common, which affect the RMSE negatively but do not necessarily have a large impact on the qualitative behavior of the model. It is to be noted that only the evaluations performed using purely simulated values are relevant for the evaluation of the effect of parameter changes, as measured values cannot be used as intermediate values when the goal is to analyze the effect of parameter changes. Therefore, in the following, the values derived from the calibration using configuration *sim_only_true*, which also showed a slightly better overall fit, were used for parameter optimization.

### 4.3. Parameter Optimization

Using the calibrator, parameter optimization was performed in regards to the heating system’s gas consumption. Therefore, the free parameters were set to their respective calibrated values and a subset of the controller parameters which were previously fixed was turned into calibratable free parameters. In order to perform a parameter optimization, however, it is necessary to modify the optimization target. To achieve this, so-called penalty-expressions like the example shown in Penalty-Expression 1 are included to refine the optimization target. The use of penalty-expressions alone, however, may lead to over fitting, when the overall system behavior is not sufficiently well defined. Optimizing purely towards a minimization of the gas consumption for example could, in the worst case, lead to a complete shutdown of the heat producer. Opposed to this, generally the overall behavior should be maintained while only improving on certain characteristics. To mitigate over fitting, the penalties from the penalty-expressions are added to the computed RMSE, hence providing stability to the optimization process. By adjusting the magnitude of the penalties, the impact of the RMSE compared to the weighting of the penalty-expression can be adjusted.

The chosen parameter mapping can be seen in Table 3. The parameter “UpperLimitParam” causes the boiler as well as the boiler pump to turn on when the temperature at the upper probe of the heating buffer storage tank falls below the specified value. When the value at the lower probe exceeds the value of “LowerLimitParam”, the boiler is turned off again. Similarly, the “ActivationTemperatureParam” causes the loading pump of the hot water circuit to turn on when the temperature at the upper probe of the hot water storage tank falls below the specified value and deactivates the loading pump when the temperature at the lower probe exceeds the value of “DeactivationTemperatureParam”. The parameter “TargetTemperatureParam” refers to the target temperature that the three-way mixing unit of the hot water circuit has to achieve and supply to the heat exchanger on the primary side. The “TargetOuputTemperatureParam” is the reference target temperature supplied to the boiler controller and was given physically reasonable bounds. The mutation and recombination rates of the GA Calibrator, during parameter optimization, were both set to 0.2, while the remaining hyperparameters were kept at their default values as described in Section 2.3. The following behavior was penalized:

- Simulated buffer flow temperature (secondary side) exceeding 95 °C (1.0 × 10^9^ penalty).
- Aggregated boiler gas consumption (aggregated gas consumption · 100 as penalty).
- Boiler state switches (number of status switches as penalty).
- Loading pump status switches (number of status switches as penalty · 10).
- Average hot water temperature being below 58 °C (1.0 × 10^9^ penalty).
- Target temperature of the three-way mixing unit (primary hot water supply to the heat exchanger) being lower than the activation temperature (1.0 × 10^9^ penalty).
- Hot water temperature falling below 45 °C (5 × 10^3^ penalty).
- Hot water temperature exceeding 75 °C (5 × 10^3^ penalty).
- Small penalty every time the hot water temperature falls below 55 °C (10 penalty each).

The last six terms were combined in a single penalty-expression ensuring that the hot water temperature remains sufficiently high, as it is measured at the outlet of the hot water storage tank and is subject to cooldown when traversing pipes in the residential building. Each penalty-expression is applied separately for each dataset.

### 4.4. Parameter Optimization Results

The following section describes the results of the parameter optimization. Additionally, we conducted separate parameter optimizations for winter and summer data. The resulting two sets of parameters were then combined for simulating the entire year, utilizing the winter parameters during the heating period and the summer parameters for the remainder of the year.

A parameter calibration run was performed. The parameter values obtained are listed in Table 4. Different calibration runs using this configuration resulted in similar parameter values, as well as similar model behavior, indicating that the calibration procedure is stable.

**Figure 8:**
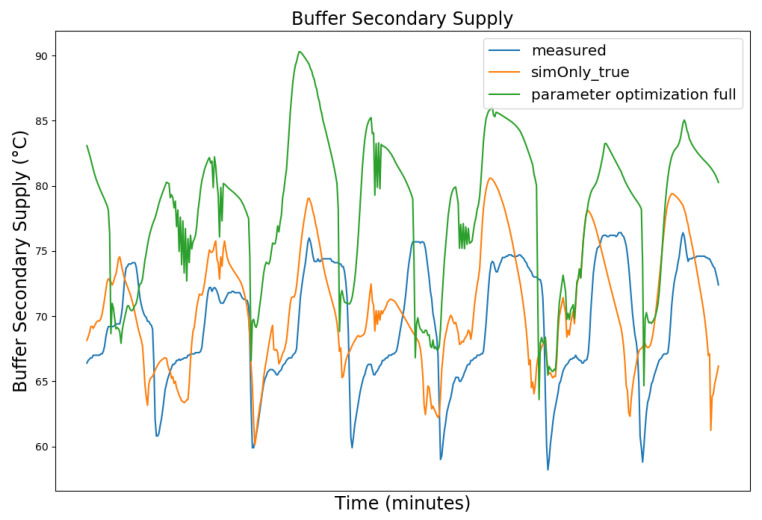
Data snapshot from 27 February 2019 05:00 a.m. to 27 February 2019 01:20 p.m. The graphs are showing the supply temperature of the heating storage buffer tank (secondary side) as a comparison of the measured data (blue), the model calibrated on the configuration *sim_only_true* with simulated values overwriting measured ones (orange), and the model after the controller parameter optimization with simulated values overwriting measured ones (green).

**Figure 9:**
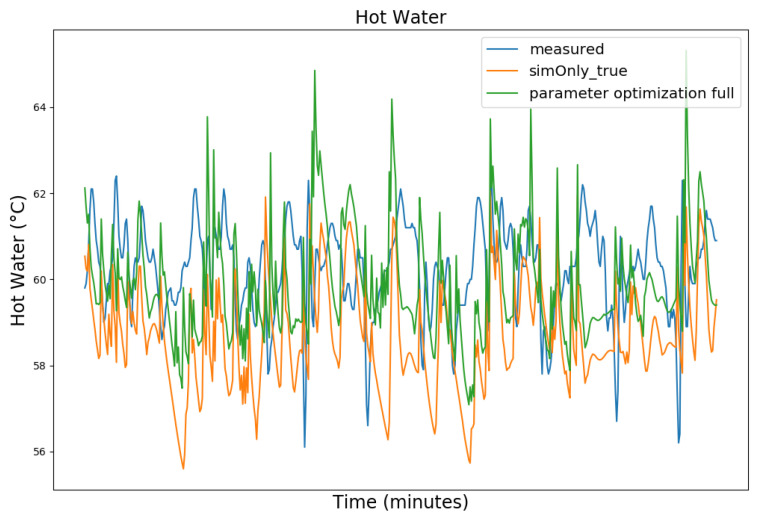
Hot water temperature at the hot water storage tank outlet. For an explanation of the graph legend, please refer to the caption of Figure 8.

**Figure 10:**
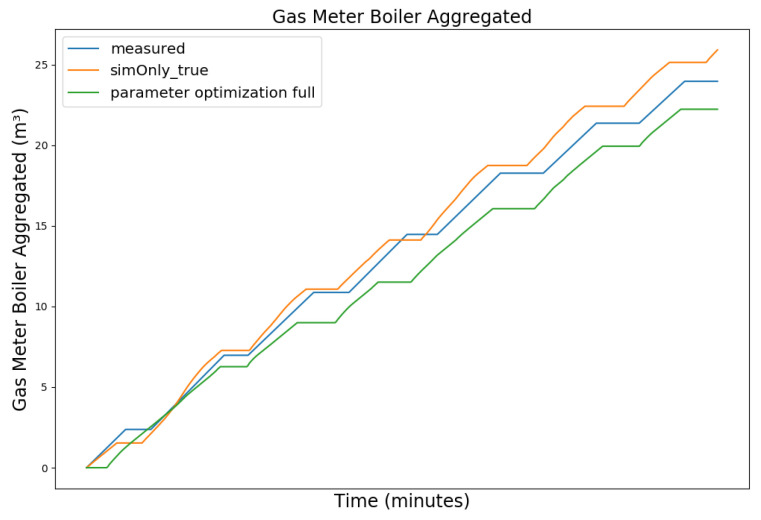
Aggregated boiler gas consumption. For an explanation of the graph legend, please refer to the caption of Figure 8.

**Figure 11:**
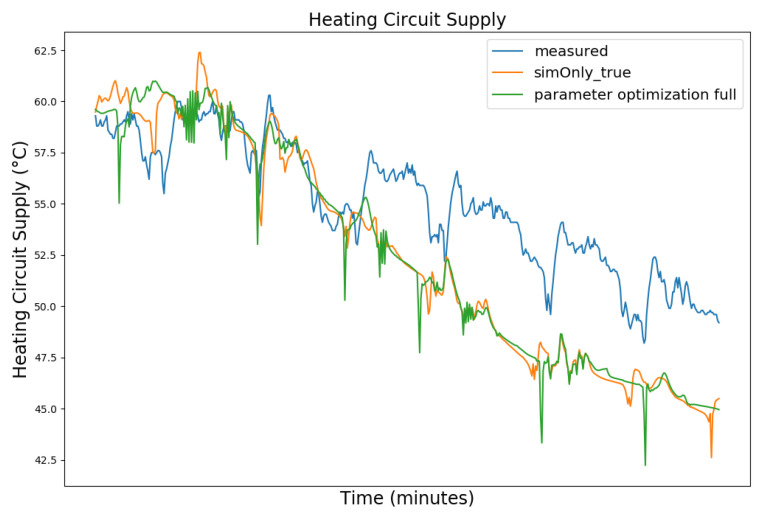
Supply temperature of the heating circuit (primary side). For an explanation of the graph legend, please refer to the caption of Figure 8.

Figure 8, Figure 9, Figure 10 and Figure 11 exemplary show the model behavior after the calibrator was used to generate values for the controller parameters for a snapshot of the winter data, as during the summer the heating circuit is shut off. The parameters were chosen so that the temperature of the storage tank was increased, causing the boiler to perform significantly less state-switches and to increase the duration of the on-state slightly, thus decreasing the off-state duration during which the boiler efficiency decreases. The elevated buffer storage tank temperature, on the other hand, reduces the amount of water requested from the storage tank to heat the hot water and the heating circuit, increases the return temperature to the boiler, and causes the boiler to reduce its power output, thus achieving a trade off between longer boiler run time in exchange for a greater overall gas saving. The heating circuit adapted to these changes automatically via its integrated three way mixing unit. Due to the coarse nature of the model, we do not expect the actual system behavior to exactly coincide with the simulated one, including the magnitude of the calculated reduced gas consumption, but to be of qualitative nature instead. Furthermore, the activation and deactivation temperatures of the loading pump in the hot water circuit were set to ensure continuous operation, mirroring the effective behavior of the physical heating system. Overall, the calibration target was met with a plausible model behavior, given the parameter changes.

Table 5 shows the metrics obtained for the boiler for the full 2019–2020 dataset.

Comparing the model behavior after parameter optimization to the calibration based on *sim_only_true*, the number of simulated state switches of the boiler decreased significantly by 19.26% while the boiler runtime increased proportionally less by only 8.07%, indicating that some of the short duration off-states were reduced. While the simulated gas consumption is still higher than the measured gas consumption, it has been reduced, as compared to the initial simulation, by 6.05%. It is to be noted that the model overestimates the relative gas consumption during summer, as the stabilizing heating circuit is turned off during this time and the hot water circuit gains relative importance, which in turn is dependent on our coarse estimation of the residents’ water consumption. While the gas consumption after the optimization is reduced during winter, it is increased during summer. Over the span of the full year, this still results in a reduction in simulated gas consumption, as the absolute required amount of gas is significantly higher during winter than during summer, but also shows that there is still room for improvement.

**Figure 12:**
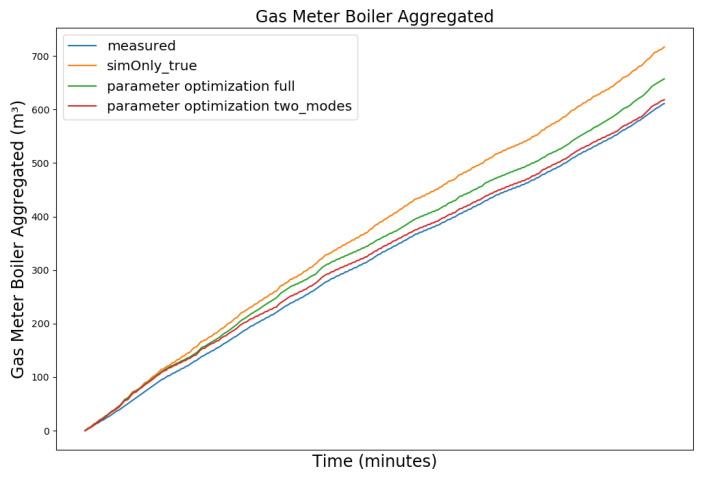
Gas consumption for a snapshot of winter data from 4 January 2020 11:19 p.m. to 11 January 2020 09:59 p.m.

**Figure 13:**
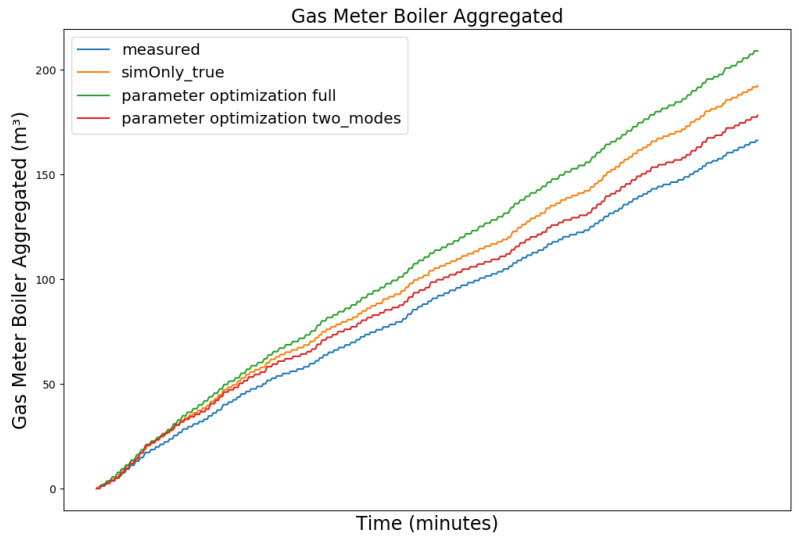
Gas consumption for a snapshot of summer data from 16 July 2019 11:59 p.m. to 23 July 2019 10:39 p.m.

Figure 12 and Figure 13 show the measured gas consumption (blue), as well as the gas consumption for the model calibrated on the configuration *sim_only_true* (orange) for the model after parameter optimization (green) for a snapshot of the winter and summer data, respectively. As can be seen, the optimized version improves the gas consumption during winter but causes an increase during summer. Since the heating circuit is shut off during summer and the hot water circuit is not able to absorb all the additional heat, the boiler’s on-cycles do not increase like they did during winter. The elevated temperatures, however, without the longer on-cycles, increase the gas consumption. Based on this observation, we have investigated the use of one set of controller parameters for the heating period and a different set of controller parameters for the remainder of the year. We therefore split the parameter optimization and performed two separate optimization runs. For the first run, we have selected two datasets with 5000 data points each for the three month winter period between 16 November 2019 to 16 February 2020 and two datasets of equal length from the three month summer period between 1 June 2019 and 31 August 2019 as training data. Finally, we evaluated the simulated gas consumption using the two sets of optimized controller parameters during the heating period and the remainder of the year, respectively. While the heating period as such is not explicitly defined by law in Germany, it is generally considered to be between 1 October to 30 April and was subsequently used for our evaluation. As can be seen in the corresponding graphs (red) of the Figure 12 and Figure 13, the controller parameters optimized based on seasonality show a reduction in the gas consumption for both the snapshot of the winter and the snapshot of the summer data, as compared to *sim_only_true*.

Table 6 shows the corresponding improvements of the parameter optimization based on seasonality, consisting of two season dependent sets of controller parameters, as compared to *sim_only_true* and the parameter optimization using one single set of controller parameter values, evaluated on the full 2019–2020 dataset. The parameter optimization based on seasonality slightly increased the number of boiler state switches by 4.98%, decreased the total boiler runtime by 11.74%, and further decreased the gas consumption by 4.1% as compared to the original parameter optimization (full year). Compared to the initial calibration, based on the configuration *sim_only_true*, the total number of state switches decreased by 15.24%, the total boiler runtime decreased by 4.62%, and the gas consumption decreased by 9.9%. The results could be improved further by defining the start and end date of the heating period based on outside temperature rather than a fixed date. However, since there is no mechanism to automatically update the controller parameters of the physical heating system, two manual controller parameter updates per year on a fixed date seemed more viable. This parameter change could be performed by a janitor, a technician, or even the residents themselves, if they have permission and access to the boiler room, as the parameter changes can be made comfortably using the displays and buttons provided by the respective controllers.

## 5. Conclusions

We have presented a system to calibrate and simulate heating system models which can be embedded into an existing data acquisition architecture. Our system allows for the preprocessing of raw data, which can then be used to automatically calibrate heating system models, which in turn can be used to simulate possible changes to controller parameter values. Further, the calibration process can be turned into a parameter optimization process through reformulation to automatically generate values for controller parameters, optimizing the system’s behavior towards a self-defined target, guided by a set of penalty-expressions. The preprocessor module and the simulator and calibrator module can be used locally or deployed as two separate microservices, which can easily be interfaced with an existing data acquisition infrastructure. For evaluation, we first selected a physical heating system retrofitted with temperature sensors and heat meters, which were connected to the existing data acquisition platform of ENER-IQ GmbH. We then built a coarse model, calibrated using the preprocessed measured data, and then used it as a base to perform parameter optimization. There were two main results. The first result was that it was possible to calibrate a coarse heating system model (see schema of Figure 2) of a 30-unit residential building, given measured data and the actual controller parameters, even though critical values like the water consumption behavior of the residents could not be measured and had to be estimated during preprocessing (see results Table 2). Based on the calibration results of the configuration *sim_only_true* we then turned some of the previously fixed controller parameters into calibratable parameters and performed a parameter optimization with the goal of reducing the boiler gas consumption. We did so by modifying the calibration target through so-called penalty-expressions, which penalized gas consumption as well as other undesirable behavior. This led to the second main result, which was a successful decrease in gas consumption compared to the initial calibration by 6.05% (see results Table 5). Since this optimization was based on a full year of data, without accounting for seasonality, further improvement was possible. For this further improvement, we split the optimization into winter and summer months and performed one optimization run for samples from each period. The resulting evaluation using the winter parameters during the heating period and the summer parameters during the non-heating period showed a further improvement as compared to the optimization based on the full year by 4.1%, resulting in a total decrease in the gas consumption by 9.9% as compared to the initial calibration (see results Table 6). Further, from our experiments we found that the model deviated from the physical system in some aspects, like the supply temperature of the heating circuit, which could not be overcome by calibration. We therefore assume that the deviation is inherent to the model. Inaccuracies like these naturally degrade the accuracy of both the calibration and the quality of the parameter optimization. While it would be of interest to verify the exact effect of the optimized parameters on the real heating system by applying them to the physical controllers, obtaining the current controller parameters and the permissions to make the parameter adjustments, along with the lack of generality, makes the associated cost unreasonably high. As noted in Section 1.2, similar to the related work, we instead focus on simulation and the methodology of parameter optimization. Overall, our results showed that our approach to model calibration and parameter optimization is viable and could be applied to a real heating system, given the required measured data can be obtained and enough resources are available to apply the optimizations to the real heating system. If the general conditions for practical use are favorable, the application of such a system could contribute to more efficient and optimized heating systems, addressing challenges in energy consumption and system performance.

Building on our work, we want to expand the application of our system to a broader range of heating systems, as well as gradually improve the model quality and the data acquisition workflow. Additionally, we aim to employ a Decision Support System (DSS), which together with the simulator and calibrator, will be embedded into a Decision Integration System (DIS) and automated by a process controller. The process controller will analyze the measured data and generate status descriptions, which will be periodically sent to the Decision Support System to generate possible suggestions for actions to be performed. The actions may be in the form of recommendations for adjustments to the controller parameters. Such actions will then be forwarded to the simulator and calibrator to perform a simulation of the recommendation. If the process controller identifies a potential improvement through a qualitative “better/worse” assessment of the resulting simulated data, the recommendation should then be forwarded to a technician for a final check and potential implementation, if the required permissions and available resources allow for it. Furthermore, the process controller will initiate periodic recalibrations of the underlying models to keep them up to date and to detect potential undocumented changes to the physical heating systems.

## Figures and Tables

**Table 1:** Overview of the available datasets with information on the number of missing data points and availability of the controller parameters.

Dataset Timeframe	No. of Missing Data Points	Availability of Controller Parameters
February 2019–February 2020	22,543 (4.29% of total dataset)	Recent controller parameters for the boiler, hot water circuit, and heating circuit available.
February 2020–February 2021	76,556 (14.53% of total dataset, one extra day due to leap year)	Boiler parameters unavailable. No recent parameters for hot water circuit and heating circuit.
February 2021–February 2022	103,818 (24.61% of total dataset)	Boiler parameters unavailable. Recent controller parameters for hot water circuit and heating circuit available.

**Table 2:** RMSE values (in °C) for the key temperatures of the model using the configurations *sim_only_false* (executed twice: (1) allowing measured values to overwrite intermediate simulated values and (2) using simulated values only as intermediate values) and *sim_only_true*.

Value (Measured Range)	*so_False* (1)	*so_False* (2)	*so_True*
Buffer Secondary Supply (49.9–77.0 °C)	2.77	6.19	6.24
Buffer Secondary Return (33.6–64.5 °C)	2.07	4.62	4.31
Boiler Supply (48.4–82.9 °C)	4.05	6.33	6.36
Boiler Return ( 37.4–71.2 °C)	2.76	6.17	6.01
Heating Circuit Supply (26.7–74.0 °C)	2.38	2.53	2.56
Heating Circuit Return (21.2–43.9 °C)	2.50	2.53	2.47
Hot Water (43.9–64.8 °C)	1.86	2.57	2.28
Circulation (32.9–58.9 °C)	1.37	2.46	1.95
Hot Water Circuit Primary Supply (42.3–75.6 °C)	2.86	3.86	3.85
Hot Water Circuit Primary Return (32.0–71.7 °C)	4.12	6.27	5.59
Hot Water Circuit Secondary Supply (47.8–68.6 °C)	3.75	6.06	5.05
Hot Water Circuit Secondary Return (8.0–57.4 °C)	2.87	3.51	3.96

**Table 3:** Parameter mapping for the parameter optimization.

Index	Portname	minValue	maxValue
0	BoilerRegulator.UpperLimitParam	0	100
1	BoilerRegulator.LowerLimitParam	0	100
2	Hotwatercircuit.ActivationTemperatureParam	0	100
3	Hotwatercircuit.DeactivationTemperatureParam	0	100
4	ThreewayMixingUnit.TargetTemperatureParam	0	100
5	BoilerRegulator.TargetOutputTemperatureParam	60	80

**Table 4:** Comparison of parameter mappings before parameter optimization (orig.) and after parameter optimization (optim.).

Portname	minValue	maxValue	Orig.	Optim.
BoilerRegulator.UpperLimitParam	0.0	100.0	58.0	75.4
BoilerRegulator.LowerLimitParam	0.0	100.0	68.0	77.17
Hotwatercircuit.ActivationTemperatureParam	0.0	100.0	57.0	33.1
Hotwatercircuit.DeactivationTemperatureParam	0.0	100.0	58.0	100.0
ThreewayMixingUnit.TargetTemperatureParam	0.0	100.0	63.0	64.06
BoilerRegulator.TargetOutputTemperatureParam	60.0	80.0	69.0	66.49

**Table 5:** Comparison of boiler metrics between the model calibrated using the configuration *sim_only_true* and the model after parameter optimization based on a full year.

Feature	Measured	*so_True*	Optim. Full Year
Total number of state switches	14,738	13,893	11,217
Total boiler runtime (min)	268,436	266,722	288,240
Aggregated gas consumption (m^3^)	19,695.04	22,283.78	20,936.15

**Table 6:** Comparison of boiler metrics between the model calibrated using the configuration *sim_only_true*, after parameter optimization based on a full year, and after the parameter optimization based on the winter-summer split.

Feature	Measured	*so_True*	Optim. Full Year	Optim. Seasonal
Total number of state switches	14,738	13,893	11,217	11,776
Total boiler runtime (min)	268,436	266,722	288,240	254,403
Aggregated gas consumption (m^3^)	19,695.04	22,283.78	20,936.15	20,078.58

## Data Availability

The datasets of the heating system presented in this article are not publicly available due to privacy reasons.

## Abbreviations

The following abbreviations are used in this manuscript:

RMSE	Root Mean Square Error
GA	Genetic Algorithm
CGA	Cyclic Genetic Algorithm
LSTM	Long Short-Term Memory
DSS	Decision Support System
DIS	Decision Integration System
PSO	Particle Swarm Optimization
DE	Differential Evolution
BO	Bayesian Optimization
HVAC	Heating, Ventilation, and Air Conditioning
AWS	Amazon Web Services
MQTT	Message Queuing Telemetry Transport
JSON	JavaScript Object Notation
IoT	Internet of Things

## Appendix A. Calibratable Parameters

For illustration purposes, Table A1, Table A2, Table A3, Table A4, Table A5, Table A6 and Table A7 show the calibratable parameters as well as the calibrated parameters for the configuration *sim_only_false*.

**Table A1:** Calibratable parameter mapping table content for the HeatingBuffer component.

Index	Portname	minValue	maxValue	Value
0	HeatingBuffer.PenetrationDepthParam	0	20.00	18.26
1	HeatingBuffer.PenetrationDistributionParam	1.50	20.00	4.98
2	HeatingBuffer.PenetrationMixingFactorParam	0	0.10	0.10
3	HeatingBuffer.RatioScalarParam	0.01	5.00	1.86
4	HeatingBuffer.MixingRatioScalarParam	0	0.10	5.5 × 10^−3^
5	HeatingBuffer.DissipationParam	5.0 × 10^−4^	5.4 × 10^−4^	5.4 × 10^−4^

**Table A2:** Calibratable parameter mapping table content for the Boiler component.

Index	Portname	minValue	maxValue	Value
6	Boiler.KpPositiveParam	0.05	0.10	0.05
7	Boiler.KiPositiveParam	0	2.00	1.51
8	Boiler.KdPositiveParam	0	2.00	0.52
9	Boiler.WindupGuardPositiveParam	0	100.00	0
10	Boiler.MinEfficiencyParam	0.05	0.75	0.05
11	Boiler.EfficiencyIncreaseParam	0.08	0.50	0.50
12	Boiler.EfficiencyDecreaseParam	0.95	0.96	0.95
13	Boiler.MaxEfficiencyParam	0.92	0.98	0.98
14	Boiler.InertiaParam	8.0 × 10^−3^	9.0 × 10^−3^	8.0 × 10^−3^
15	Boiler.BoilerCooldownParam	0.99	1.00	1.00
16	Boiler.BoilerAnnealScalarParam	1.0 × 10^3^	1.0 × 10^9^	1.0 × 10^9^

**Table A3:** Calibratable parameter mapping table content for the HeatingCircuit component.

Index	Portname	minValue	maxValue	Value
17	HeatingCircuit.KpParam	0	2.00	0.38
18	HeatingCircuit.KiParam	0	2.00	0.65
19	HeatingCircuit.KdParam	0	2.00	0.56
20	HeatingCircuit.WindupGuardParam	0	100.00	0
21	HeatingCircuit.BuildingInertiaParam	3.00	100.00	29.76
22	HeatingCircuit.TenvOffsetParam	0	5.00	1.15

**Table A4:** Calibratable parameter mapping table content for the HotWaterBuffer component.

Index	Portname	minValue	maxValue	Value
23	HotWaterBuffer.PenetrationDepthParam	0	20.00	3.89
24	HotWaterBuffer.PenetrationDistributionParam	1.50	20.00	20.00
25	HotWaterBuffer.PenetrationMixingFactorParam	0	0.10	0
26	HotWaterBuffer.RatioScalarParam	0.01	5.00	4.28
27	HotWaterBuffer.MixingRatioScalarParam	0	0.10	5.72 × 10^−3^
28	HotWaterBuffer.TwzOffsetParam	2.50	5.50	3.63
29	HotWaterBuffer.DissipationParam	0	0.01	3.20 × 10^−3^
30	HotWaterBuffer.RequestWaterInhabitantsCutoffParam	0	0.05	0.05

**Table A5:** Calibratable parameter mapping table content for the HeatExchanger component.

Index	Portname	minValue	maxValue	Value
31	HeatExchanger.KAParam	1.70	1.90	1.90
32	HeatExchanger.DefaultDtlnParam	0.10	10.00	4.41
33	HeatExchanger.TempAnnealScalarParam	0.90	1.00	0.97

**Table A6:** Calibratable parameter mapping table content for the ThreewayMixingUnit component.

Index	Portname	minValue	maxValue	Value
34	ThreewayMixingUnit.KpParam	0	2.00	2.00
35	ThreewayMixingUnit.KiParam	0	2.00	0
36	ThreewayMixingUnit.KdParam	0	2.00	0
37	ThreewayMixingUnit.WindupGuardParam	0	100.00	60.99

**Table A7:** Calibratable parameter mapping table content for the HotWaterChargingPump component.

Index	Portname	minValue	maxValue	Value
38	HotWaterChargingPump.KpParam	0	2.00	0.54
39	HotWaterChargingPump.KiParam	0	2.00	0
40	HotWaterChargingPump.KdParam	0	2.00	1.36
41	HotWaterChargingPump.WindupGuardParam	0	100.00	100.00
